# Field Electron Emission from Crumpled CVD Graphene Patterns Printed via Laser-Induced Forward Transfer

**DOI:** 10.3390/nano12111934

**Published:** 2022-06-06

**Authors:** Maxim Komlenok, Nikolay Kurochitsky, Pavel Pivovarov, Maxim Rybin, Elena Obraztsova

**Affiliations:** Prokhorov General Physics Institute of the Russian Academy of Sciences, st. Vavilov 38, 119991 Moscow, Russia; kuronick@mail.ru (N.K.); p_pivovarov@hotmail.com (P.P.); rybmaxim@gmail.com (M.R.); elobr@kapella.gpi.ru (E.O.)

**Keywords:** field electron emission, crumpled CVD graphene, laser-induced forward transfer, laser processing, Raman spectroscopy

## Abstract

A new approach to the fabrication of graphene field emitters on a variety of substrates at room temperature and in an ambient environment is demonstrated. The required shape and orientation of the graphene flakes along the field are created by the blister-based laser-induced forward transfer of CVD high-quality single-layer graphene. The proposed technique allows the formation of emitting crumpled graphene patterns without losing the quality of the initially synthesized graphene, as shown by Raman spectroscopy. The electron field emission properties of crumpled graphene imprints 1 × 1 mm^2^ in size were studied. The transferred graphene flakes demonstrated good adhesion and emission characteristics.

## 1. Introduction

The transition from bulk to low-dimensional materials has opened up new opportunities in the creation of new electronic elements and the modification of existing ones, particularly field emitters, due to their adjustable band gap and promising electronic properties [1,2,3,4]. The interest in these materials has increased significantly due to the discovery of the unique properties of graphene [5]. Currently, 2D graphene sheets are the subject of active research for field emission (FE) applications [6,7,8,9,10,11,12]. Graphene has high carrier mobility, as well as excellent electrical and thermal conductivity. In addition, graphene offers the possibility of obtaining a high aspect ratio and a high carrier density due to the presence of active sharp edges in atomic size and confined defects, which are favorable for the enhancement of the barrier field, resulting in a high FE current associated with a low turn-on potential. All these factors make graphene a promising base material for the creation of field emitters. On the other hand, the excellent mechanical properties and chemical stability of graphene make the use of these emitters particularly relevant for the creation of flexible displays and miniaturized X-ray tubes [13,14,15,16,17].

Graphene field emitters can be produced by different techniques, such as micromechanical exfoliation [18,19], chemical vapor deposition (CVD) [20,21,22], electrophoretic deposition [23], the reduction from graphene oxide [24], etc. When creating emitters based on graphene, difficulties arise with the orientation of the graphene sheets along the field [23,25,26]. Various methods have been used to overcome this problem, such as the synthesis of vertically oriented graphene [25], screen printing [27], or the deposition of thin graphene–polymer composite films [23]. The listed methods are quite laborious or introduce pollution during the transfer of the graphene. We propose to apply the laser-induced forward transfer (LIFT) technique for this purpose, which allows the transfer of materials in a liquid or solid phase for different optical and electronic applications [28,29,30]. This approach has already been successfully used for the precise transfer of carbon nanomaterials, such as single-walled carbon nanotubes (CNT), diamond nanoparticles, and graphene [31,32,33,34,35]. In particular, the LIFT technique has been utilized for printing CNT FE cathodes via their partial evaporation [36]. Recently, we showed that blister-based (BB) LIFT makes it possible to print crumpled graphene from the substrate with CVD high-quality single-layer graphene without losing its quality [37].

The method of BB-LIFT consists of local laser surface evaporation of a thin absorbing layer, such as metal, covering a transparent substrate, usually quartz. As a result, a blister is formed at the interface between the quartz and the metal film, which pushes the transferred material lying on the metal from the irradiated substrate (donor) to the receiving substrate (acceptor). The obvious advantages of the proposed approach are the variety of substrates available for the emitters and their arbitrary shape. Here, we report the results of the BB-LIFT technique applied to the printing of pixels of crumpled graphene and of the emission test of the field-emission cathodes fabricated in this way.

## 2. Materials and Methods

For donor-sample preparation, an aluminum film was deposited in a vacuum chamber (10^−5^ mbar) on the surface of a polished quartz substrate from a molybdenum boat heated to 900 °C by the current passing through it (resistive heating). The thickness of the aluminum film was measured by the white-light interferometer Zygo NewView 5000 (USA) and amounted to 1900 nm. A single-layer polycrystalline graphene film grown on copper foil by CVD technique was transferred to the metal surface of the donor sample by a standard wet method using poly(methyl methacrylate) as a supporting polymer, which was chemically removed after the transfer [38].

**Figure 1:**
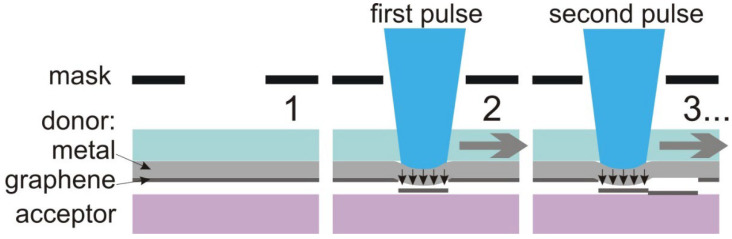
Sketch of the BB-LIFT technique used to print a graphene emitter. The large arrows indicate the shift of the donor and acceptor relative to the laser beam.

The BB-LIFT procedure included the use of a KrF excimer laser (wavelength 248 nm, pulse duration 20 ns). The scheme of irradiation is shown in Figure 1. The receiving substrate, the so-called acceptor, consisted of the silicon plate (thickness of 400 µm) and a copper film (thickness of 500 nm) deposited on it by the same technique as the aluminum film described above. The acceptor substrate was placed in contact with the donor to best match the patterns on the acceptor to the shape of the laser spot and improve the quality of the transferred graphene flakes, as reported previously [37]. The laser beam was focused onto the square spot, which was 200 × 200 μm^2^, on the aluminum film. The donor and acceptor were mounted together onto the translational stage and were moved sequentially with a step equal to the spot size to transfer the area of 1 × 1 mm^2^. Two types of acceptor sample were produced: one and three passes over the area. For the multipass transfer of graphene flakes to the acceptor, the donor substrate was shifted relative to the acceptor to the new position and irradiated again.

The quality of graphene on the donor and acceptor substrates was characterized using Raman spectroscopy (Horiba LabRAM HR Evolution spectrometer (Japan) with λ = 532 nm) and scanning electron microscopy (SEM) (TESCAN Mira 3 (Czech)). The surface morphology was also analyzed using an Axiotech 25HD optical microscope (Carl Zeiss (Germany)).

FE properties of the two acceptors were studied in a flat vacuum diode configuration at 10^−6^ mbar and room temperature. The acceptor substrate (cathode) was installed on a holder parallel to the anode at a distance *d* = 400 µm under it. A DC voltage was applied between the cathode and anode, causing the emission of electrons from the graphene flakes. As a result, the current–voltage (I/U) dependences were measured.

## 3. Results and Discussion

**Figure 2:**
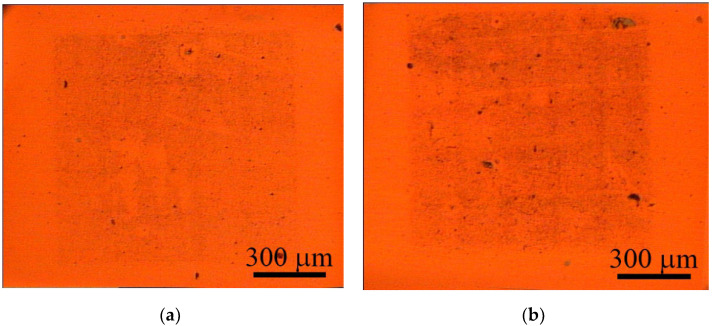
Optical images of complete transferred areas on receiving substrates after single (**a**) and triple (**b**) laser exposure.

**Figure 3:**
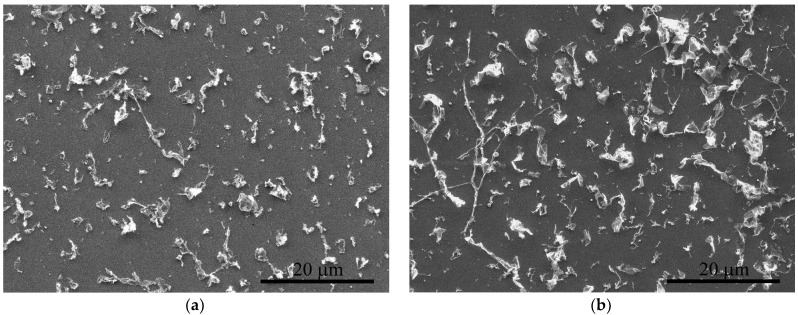
SEM images of characteristic printed regions after single (**a**) and triple (**b**) laser exposure.

The working range of laser fluence for graphene flake transfer determined in our previous work [37] is 1.2–2.8 J/cm^2^. In this work, we used the fluence of 2.3 J/cm^2^, which is the most promising value for imprint quality. The lower values did not cause the complete transfer of the graphene from the donor substrate. The higher values could lead to structural damage to the graphene due to its overheating. The result of the BB-LIFT of the single-layer graphene film after one and three passes is shown in Figure 2. One can see the clear square shape of the transferred area of 1 × 1 mm^2^ with a relatively uniform distribution of graphene flakes on the acceptor substrate. The SEM images (Figure 3) indicate that after the laser exposure and transfer, the polycrystalline graphene film broke into separate micron-sized flakes. The surface coverage density of the transferred carbon nanomaterial was larger in the case of multipass irradiation, which was confirmed by optical (Figure 2) and electron (Figure 3) microscopy. To estimate the percentage of surface coverage, we analyzed the SEM images with the graphics software previously used to calculate the amount of multilayer graphene on the surface [39]. After converting the SEM image to the black–white format, each pixel was represented by ‘0’ and ‘1’, and the percentage of surface coverage was calculated as 10.6 % for the single-pass and 17.9 % for the three-pass irradiated areas. The non-threefold difference was most likely associated with the possibility of overlapping the flakes on the acceptor substrate after multiple transfers.

**Figure 4:**
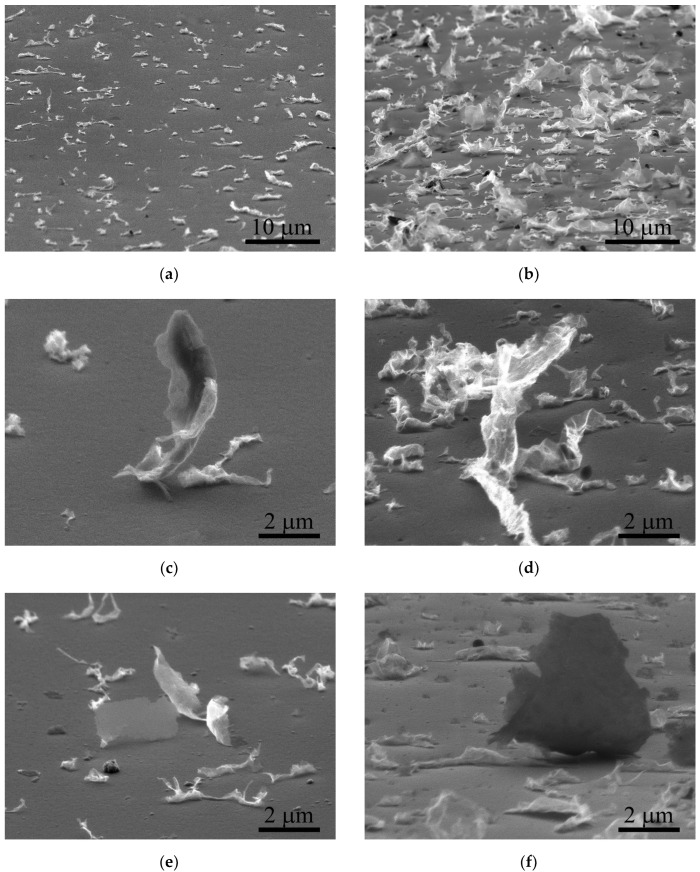

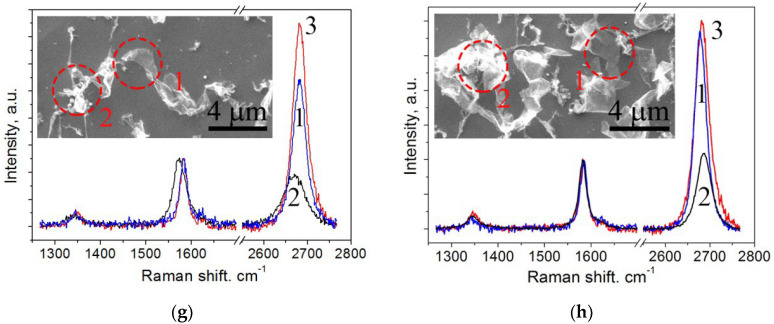
The side-view SEM images of characteristic-pattern regions and single elements with their Raman spectra after single (**a**,**c**,**e**,**g**) and triple (**b**,**d**,**f**,**h**) laser exposure. Blue curves in the Raman spectra correspond to the transferred flat graphene fragments (area 1 in the inset), black curves to the transferred crumpled graphene (area 2 in the inset), and red curves to the original graphene before transfer (3).

For a more detailed analysis of the morphology of the transferred graphene flakes and their contact with the acceptor substrate, we used SEM images with inclined (70°) views. Essentially, in the transferred carbon material, there were separate, highly crumpled sections of film, both of which were in strong contact with the substrate and were located at a large angle from the surface of the copper film, rising steeply (up to ~10 μm) and touching it in only a few points (Figure 4). A possible cause of the creasing was the free flight of the graphene from the donor to the acceptor substrate due to its tendency to reduce the surface energy in the free state. The analysis of the transferred areas by Raman spectroscopy showed that the original structure of the graphene film was retained, since the D peak (1345 cm^−1^) on the flakes was an order of magnitude lower than the G peak (1580 cm^−1^), but according to the shape of the transferred graphene from the donor to the acceptor, two varieties were highlighted, which were clearly distinguishable in the Raman spectra. The first type was areas that were weakly visible in the optical images, and corresponded to the Raman spectra of the graphene monolayer. The Raman spectra of these areas had a high ratio of 2D (2670 cm^−1^) to G peak intensities (more than 2) and narrow 2D peaks (less than 35 cm^−1^). We suppose that in these regions, the graphene monolayer was transferred from the donor to the acceptor without morphological changes, in the form of so-called flat graphene. The main part of the transferred material, which was already clearly visible by optical methods (dark areas), was crumpled graphene, with the ratio of the intensities of the 2D and G peaks (Figure 4) corresponding to two- and three-layer graphene, which explains the high optical contrast of these areas. However, the width of the 2D peak for this graphene material was closer to the initial width of the single-layer graphene of 32 cm^−1^ than to the 50 cm^−1^ typical of flat multilayer graphene films. In our opinion, the observed features in the Raman spectra are explained precisely by the wrinkling of the initial monolayer graphene film and by the scattering of phonons with weak interlayer interaction under these conditions. The Raman spectra also indicate the presence of bends and stresses, which are expressed in the shift in the 2D peak [40].

**Figure 5:**
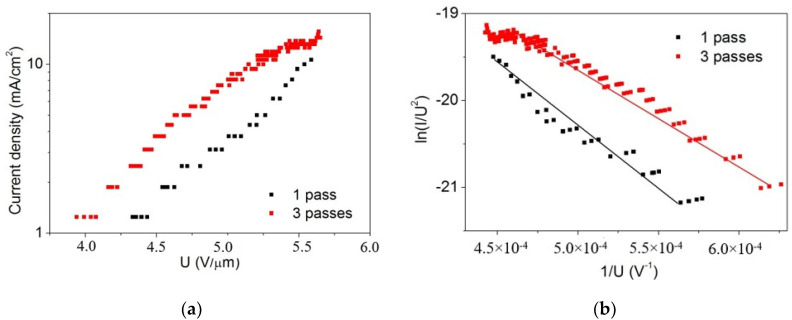
(**a**) Field emission current density versus the electric field and (**b**) FN plot for the printed graphene cathodes after single and triple laser exposure.

The dependencies of the emission current density on the electric field for the single- and three-passed patterns are presented in Figure 5a. The threshold field *E_thr_* at the current density *J* = 1 mA/cm^2^ occurred at 4.4 and 4 V/µm for the single- and three-passed patterns, respectively. Due to the higher graphene flake density, the emission current was larger for the three-passed area over the entire range of applied voltage. The observed differences in emission characteristics were previously reported for CNT films with different thicknesses [18] and lengths of nanotubes [41]. The maximum current densities *J_max_* for the single- and three-passed patterns were 10.6 and 15.6 mA/cm^2^, respectively. It should be mentioned that the ratio *J_max_*_1_*/J_max_*_3_ = 1.5 correlated with the ratio of the surface coverage densities of the graphene flakes 17.9/10.6 = 1.7. The current–voltage characteristics of the single- and three-passed patterns in the Fowler–Nordheim (FN) coordinates (ln (I/U^2^) vs 1/U) are presented in Figure 5b. The field enhancement factor *β* can be calculated from the FN plots using the following equation *β* = −*bϕ*^3/2^*/k_FN_*, where *b* = 6.83 × 10^3^ eV^−3/2^ V^−1^ µm, *ϕ* is the work function of the graphite (5 eV), and *k_FN_* is the slope of the FN plot [9]. For one- and three-pass irradiation, the field enhancement factor was calculated to be 1900 and 3300, respectively. Although our deposited graphene flakes did not cover the entire surface, the emission characteristics (*E_thr_* = 4 V/µm, *β* = 3300) were comparable with those of other fabrication methods (*E_thr_* = 1.5 V/µm, *β* = 3000 [22], 3.09, 3200 [42], and 3.4, 1781 [43]). Further research will be focused on obtaining the maximum emission efficiency by selecting the optimal density of the deposited crumpled graphene, i.e., choosing a balance between the number of emitters and the screening effect [44] that increases with their high densities.

## 4. Conclusions

A new approach to the fabrication of graphene-based field emitters was developed. The technology demonstrated in this study is simple and based on the BB-LIFT of CVD high-quality single-layer graphene from the donor substrate to the acceptor, which results in the formation of emitting crumpled graphene pixels. The quality of the transferred graphene was maintained and corresponded to that of the single-layer graphene folded two or three times, as shown by Raman spectroscopy. The electron field emission properties of the crumpled graphene imprints 1 × 1 mm^2^ in size were tested and demonstrated Fowler–Nordheim behavior. The measured emission characteristics were comparable with those of other fabrication methods, despite the transferred graphene flakes covering only 18% of the entire surface. Further research will be aimed at obtaining the optimal density and uniformity of graphene pattern-emitting electrons.

## Data Availability

The data are available upon request from the authors.

## References

[1] Bonard J.-M., Kind H., Stöckli T., Nilsson L.-O. (2001). Field emission from carbon nanotubes: The first five years. Solid State Electron..

[2] Perales-Martinez I.A., Velásquez-García L.F. (2019). Fully 3D-printed carbon nanotube field emission electron sources within-plane gate electrode. Nanotechnology.

[3] Patra A., More M.A., Late D.J., Rout C.S. (2021). Field emission applications of graphene-analogous two-dimensional materials: Recent developments and future perspectives. J. Mater. Chem. C.

[4] Kianinia M., Xu Z.-Q., Toth M., Aharonovich I. (2022). Quantum emitters in 2D materials: Emitter engineering, photophysics, and integration in photonic nanostructures. Appl. Phys. Rev..

[5] Novoselov K.S., Geim A.K., Morozov S.V., Jiang D., Zhang Y., Dubonos S.V., Grigorieva I.V., Firsov A.A. (2004). Electric Field Effect in Atomically Thin Carbon Films. Science.

[6] Koh A.T.T., Foong Y.M., Pan L., Sun Z., Chua D.H.C. (2012). Effective large-area free-standing graphene field emitters by electrophoretic deposition. Appl. Phys. Lett..

[7] Guo Y., Guo W. (2013). Electronic and Field Emission Properties of Wrinkled Graphene. J. Phys. Chem. C.

[8] Wang Y., Yang Y., Zhao Z., Zhang C., Wu Y. (2013). Local electron field emission study of two-dimensional carbon. Appl. Phys. Lett..

[9] Chen L., Yu H., Zhong J., Song L., Wu J., Su W. (2017). Graphene field emitters: A review of fabrication, characterization and properties. Mater. Sci. Eng. B.

[10] Shao X., Srinivasan A., Ang W.K., Khursheed A. (2018). A high-brightness large-diameter graphene coated point cathode field emission electron source. Nat. Commun..

[11] Wang Q., Zhang Z., Liao Q., Kang Z., Zhang Y. (2018). Enhanced field emission properties of graphene-based cathodes fabricated by ultrasonic atomization spray. RSC Adv..

[12] Ahsan R., Sakib M.A., Chae H.U., Kapadia R. (2020). Performance Limits of Graphene Hot Electron Emission Photoemitters. Phys. Rev. Appl..

[13] Verma V.P., Das S., Lahiri I., Choi W. (2010). Large-area graphene on polymer film for flexible and transparent anode in field emission device. Appl. Phys. Lett..

[14] Hwang J.O., Lee D.H., Kim J.Y., Han T.H., Kim B.H., Park M., No K., Kim S.O. (2011). Vertical ZnO nanowires/graphene hybrids for transparent and flexible field emission. J. Mater. Chem..

[15] Lahiri I., Verma V.P., Choi W. (2011). An all-graphene based transparent and flexible field emission device. Carbon.

[16] Jeong H.J., Jeong H.D., Kim H.Y., Kim S.H., Kim J.S., Jeong S.Y., Han J.T., Lee G.W. (2012). Flexible field emission from thermally welded chemically doped graphene thin films. Small.

[17] Iwai Y., Muramatsu K., Tsuboi S., Jyouzuka A., Nakamura T., Onizuka Y., Mimura H. (2013). X-ray tube using a graphene flower cloth field emission cathode. Appl. Phys. Express.

[18] Lee S.W., Lee S.S., Yang E.H. (2009). A study on field emission characteristics of planar graphene layers obtained from a highly oriented pyrolyzed graphite block. Nanoscale Res. Lett..

[19] Nakakubo K., Asaka K., Nakahara H., Saito Y. (2012). Evolution of field electron emission pattern from multilayered graphene induced by structural change of edge. Appl. Phys. Express.

[20] Behura S.K., Nayak S., Yang Q.Q., Hirose A., Jani O. (2016). Chemical vapor deposited few-layer graphene as an electron field emitter. J. Nanosci. Nanotechnol..

[21] Yusop M.Z., Kalita G., Yaakob Y., Takahashi C., Tanemur M. (2014). Field emission properties of chemical vapor deposited individual graphene. Appl. Phys. Lett..

[22] Kleshch V.I., Bandurin D.A., Serbun P., Ismagilov R.R., Lutzenkirchen-Hecht D., Muller G., Obraztsov A.N. (2018). Field electron emission from CVD nanocarbon films containing scrolled graphene structures. Status Solidi B.

[23] Wu Z.S., Pei S.F., Ren W.C., Tang D.M., Gao L.B., Liu B.L., Li F., Liu C., Cheng H.M. (2009). Field emission of single-layer graphene films prepared by electrophoretic deposition. Adv. Mater..

[24] Jeong H.J., Kim H.Y., Jeong H.D., Jeong S.Y., Han J.T., Lee G.W. (2012). Arrays of vertically aligned tubular-structured graphene for flexible field emitters. J. Mater. Chem..

[25] Qian M., Feng T., Ding H., Lin L.F., Li H.B., Chen Y.W., Su Z. (2009). Electron field emission from screen-printed graphene films. Nanotechnology.

[26] Wang W.L., Qin X.Z., Xu N.S., Li Z.B. (2011). Field electron emission characteristic of graphene. J. Appl. Phys..

[27] Malesevic D.A., Kemps R., Vanhulsel A., Chowdhury M.P., Volodin A., Van Haesendonck C. (2008). Field emission from vertically aligned few-layer praphene. J. Appl. Phys..

[28] Serra P., Piqué A. (2019). Laser-Induced Forward Transfer: Fundamentals and Applications. Adv. Mater. Technol..

[29] Delaporte P., Alloncle A.-P. (2016). [INVITED] Laser-induced forward transfer: A high resolution additive manufacturing technology. Opt. Laser Technol..

[30] Papazoglou S., Zergioti I. (2017). Laser Induced Forward Transfer (LIFT) of nano-micro patterns for sensor applications. Microelectron. Eng..

[31] Arutyunyan N.R., Komlenok M.S., Kononenko T.V., Dezhkina M.A., Popovich A.F., Konov V.I. (2019). Printing of single-wall carbon nanotubes via blister-based laser-induced forward transfer. Laser Sci..

[32] Dezhkina M.A., Komlenok M.S., Pivovarov P.A., Rybin M.G., Arutyunyan N.R., Popovich A.F., Obraztsova E.D., Konov V.I. (2020). Blister-based laser-induced forward transfer of 1D and 2D carbon nanomaterials. J. Phys. Conf. Ser..

[33] Komlenok M.S., Kudryavtsev O.S., Pasternak D.G., Vlasov I.I., Konov V.I. (2021). Blister-Based Laser-Induced Forward Transfer of Luminescent Diamond Nanoparticles. Phys. Status Solidi A.

[34] Komlenok M.S., Kudryavtsev O.S., Pasternak D.G., Vlasov I.I., Konov V.I. (2021). Laser printing of diamond nanoparticles with luminescent SiV centers. Comput. Opt..

[35] Smits E.C.P., Walter A., de Leeuw D.M., Asadi K. (2017). Laser induced forward transfer of graphene. Appl. Phys. Lett..

[36] Chang-Jian S.K., Ho J.R., Cheng J.J., Sung C.K. (2006). Fabrication of carbon nanotube field emission cathodes in patterns by a laser transfer method. Nanotechnology.

[37] Komlenok M.S., Pivovarov P.A., Dezhkina M.A., Rybin M.G., Savin S.S., Obraztsova E.D., Konov V.I. (2020). Printing of Crumpled CVD Graphene via Blister-Based Laser-Induced Forward Transfer. Nanomaterials.

[38] Rybin M.G., Islamova V.R., Obraztsova E.A., Obraztsova E.D. (2018). Modification of graphene electronic properties via controllable gas-phase doping with copper chloride. Appl. Phys. Lett..

[39] Kondrashov I., Komlenok M., Pivovarov P., Savin S., Obraztsova E., Rybin M. (2021). Preparation of Copper Surface for the Synthesis of Single-Layer Graphene. Nanomaterials.

[40] Ferralis N. (2010). Probing mechanical properties of graphene with Raman spectroscopy. J. Mater. Sci..

[41] Sveningsson M., Morjan R.E., Nerushev O.A., Campbell E.E., Malsch D., Schaefer J.A. (2004). Highly efficient electron field emission from decorated multiwalled carbon nanotube films. Appl. Phys. Lett..

[42] Jeong H.J., Jeong H.D., Kim H.Y., Jeong S.Y., Han J.T., Lee G.W. (2013). Self-organized graphene nanosheets with corrugated, ordered tip structures for high-performance flexible field emission. Small.

[43] Deng J.H., Wu S.L., Yang Y.M., Zheng R.T., Cheng G.A. (2013). Fabricating vertically aligned ultrathin graphenenanosheets without any catalyst using rf sputtering deposition. Nucl. Instrum. Methods Phys. Res. Sect. B Beam Interact. Mater. At..

[44] Yang K., Liu J., Jiang R., Gong Y., Zeng B., Yang J., Chi F., Liu L. (2020). Maximizing the Field Emission Performance of Graphene Arrays. Nanomaterials.

